# The Effect of *Pimpinella Anisum* and *Origanum Vulgare* Extracts Against *Streptococcus Sanguinis*, *Streptococcus Mutans*, and *Streptococcus Salivarius*

**DOI:** 10.30476/DENTJODS.2021.85691.1145

**Published:** 2022-06

**Authors:** Fatemeh Lavaee, Armin Moqadas, Farzan Modarresi, Massoumeh Nowrouzi

**Affiliations:** 1 Oral and Dental Disease Research Center, Dept. of Oral and Maxillofacial Medicine, Shiraz University of Medical Sciences, Shiraz, Iran; 2 Undergraduate Students, Student Research Committee, School of Dentistry, Shiraz University of Medical Sciences, Shiraz, Iran; 3 Dept. of Microbiology, School of Medicine, Jahrom University of Medical Sciences, Jahrom Iran; 4 Postgraduate, Dept. of Periodontics, School of Dentistry, Shiraz University of Medical Sciences, Shiraz, Iran

**Keywords:** Pimpinella, Origanum, Streptococcus sanguis, Streptococcus mutans, Streptococcus salivarius

## Abstract

**Statement of the Problem:**

There are global efforts for introducing a new herbal antimicrobial agent with minimal side effects. There are some reports about the antimicrobial properties of *Pimpinella anisum* and *Oregano Vulgare*.

**Purpose:**

In this study, the antimicrobial properties of *Pimpinella anisum* and *Oregano Vulgare* have been assessed.

**Material and Method:**

In this experimental *in vitro* study, the dental plaque samples were collected from children aged 3 to 5 years old who were referred to a private dental office with diagnosis of dental caries. After determination of the bacterial colonies of *Streptococcus sanguinis*, *Streptococcus mutans* and *Streptococcus salivarius*, the minimum inhibitory concentration (MIC) and minimum bactericidal concentration (MBC) of ethanolic and methanolic extracts of *Pimpinella anisum* and *Oregano vulgare* were measured by macrodilution and microdilution methods.

**Results:**

The mean MIC and MBC of *Pimpinella anisum* extract and *Oregano vulgare* extract and their combination against *Streptococcus mutans*, *Streptococcus sanguinis*, and *Streptococcus salivarius* were statistically different (*p*< 0.001). The combination of these extracts showed the lowest MIC and MBC.

**Conclusion:**

Hydroalcoholic extracts of the *Pimpinella anisum* and *Oregano Vulgare* were effective antibacterial agent against *Streptococcus mutans*, *Streptococcus salivarius*, and *Streptococcus sanguinis* so the combination of these two extracts showed the highest antibacterial properties on all the bacteria evaluated.

## Introduction

The mouth environment can support the dental plaque formation [ 1 ]. Temperature, pH, saliva, and oxidation-reduction (redox) reactions are the main factors related to plaque development [ 2 - 4 ]. Dental plaque is a bacterial biofilm, which is formed on different surfaces in the mouth. Dental plaque is the main causative factor for dental caries and periodontal diseases. Biofilm generation first starts with pellicle formation. The loose attachment of some bacteria creates micro colonies and ultimately, biofilm maturation can cause dental pathologies [ 5 - 6 ]. The development of a biofilm allows aggregation of cell colonies, which are increasingly resistant to antibiotics [ 7 - 8 ]. There are many different bacteria responsible for biofilms formation, including gram-positive and gram-negative species [ 4 , 8 - 9 ]. *Origanum vulgare* (O.vulgare) is a popular species of *Origanum* from the mint family (Lamiaceae) [ 10 ]. It is native to western, South-western Eurasia, and the Mediterranean region. In Austrian folk medicine, *Oregano* was used as a tea or as an ointment for gastrointestinal, respiratory tract, and nervous system diseases [ 10 ]. Over 60 different compounds have been extracted. Carvacrol and thymol are dominant compounds (80%) [ 11 ]. The antibacterial activity of carvacrol against several bacteria strains such as *Escherichia coli* and Bacillus cereus has been reported [ 12 ]. Natural biocidal agents such as thymol can decrease the possibility of bacterial resistance to common antibiotics such as penicillin [ 13 ].

*Pimpinella anisum* (P. anisum), an aromatic plant from Umbethe lliferae family, has been prescribed as a carminative, galactagogue, and disinfectant, in Iranian traditional medicine [ 14 ]. The main component of the oil is anethole (80-90%) [ 15 ]. Anethole has potent antimicrobial properties, against bacteria, yeast, and fungi [ 16 ]. Anise essential oil has antiviral, antibacterial, antioxidant and anti carcinogenic properties and antifungal activity [ 13 ].

At present, due to the indiscriminate use of antibiotics, different bacteria have developed drug resistance, so new researches are conducted to introduce novel antibacterial agents. Herbal agents or their ingredients are broadly evaluated [ 7 ]. Considering the properties and ingredients of P.anisum and O.vulgare, we decided to investigate their antibacterial effects and if possible introduce a new substance with antimicrobial activity with few side effects.

Sarac and Ugur [ 17 ] showed that the essential oils of *Origanum onites* L., O.vulgare were effective against some multiple antibiotic-resistant bacteria. Kermanshah *et al*. [ 18 ] showed that P.anisum had a growth inhibitory effect against *Streptococcus mutans* (S.mutans) and Lactobacillus rhamnosus. According to this study and other similar researches, and the antibacterial effect of these two herbal extracts, we decided to investigate the synergistic antibacterial effect of these extracts against dental plaque bacteria.

## Materials and Method

In this experimental *in vitro* study, the dental plaque samples were collected from children aged 3-5 years old who were referred to a private dental office with diagnosis of dental caries.

### Pimpinella Plant Extraction

Hydroalcoholic extraction of P.anisum and O.vulgare was obtained by “maceration” method. After preparing air-dried P.anisum, 50gr of its powder was weighted by a digital balance (Sartorius, Germany) and mashed. 1500cc of the solvent (half ethanol and half water) was added to them and shaken (IKA, Germany) for 48h and 90 cycles per minute until the composition reached homogeneity. The solution was filtered (Sartorius, Germany) and the solvent was vaporized by using a rotary evaporator (KNF, USA). The sterile extract was kept in the refrigerator for microbial evaluations.

### Oregano Plants Extract

The plant was mixed with the solvent solution (20% methanol: 80% distilled water) by a ratio of 1:3(1mg powder+ 3ml solvent), and the mixture was uniformed by an electric blender for 30 minutes at room temperature. The solution was filtered for providing a transudation solution. It was dried using an incubator (Binder, Germany) at 50°C for 24 hours and the product was kept in a dry place until used. The ethanolic and methanolic extracts of P.anisum and O.vulgare were prepared. 

### Dental plaque sampling

The samples were collected from 3-5 years old children with dental caries. Dental caries was determined by cavitation on the tooth surface and evaluation of bitewing radiographs [ 20 ]. The white spot lesions and developmental grooves were excluded. A written consent form was obtained from the parents of the participants. The Ethics committee of Shiraz University of Medical Sciences has approved this study (IR.SUMS. REC. 1396.S160); also this evaluation has been conducted according to the *Declaration of Helsinki* (1975).

The participants did not have any systemic disease, especially one that could affect the rate of dental caries. Samples were taken with a sterile toothpick from dental caries. The toothpick samples were kept in 1.0-mL reduced transport fluid vials for other processes. The plaque samples dilutions were plated onto MM10-sucrose agar [ 21 ]. After 3 days of anaerobic incubation (85% N_2_, 10% CO_2_, and 5% H_2_), the colonies were presumed to be *Streptococcus sanguinis* (S.sanguinis) and s.mutans, and *Streptococcus salivarius* (S. salivarius) was selected according to their colony morphology from MM10-sucrose agar [ 22 - 23 ].

### Polymerase Chain Reaction (PCR)

**Figure 1:**
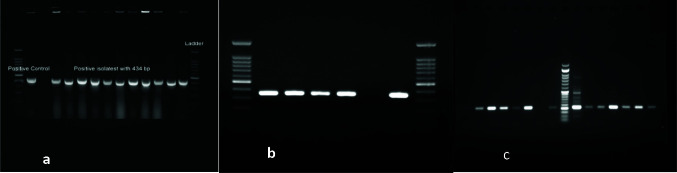
**a:** Polymerase chain reaction (PCR) amplification of patient isolated *Streptococcus mutans* species in this study. The electrophoresis agarose gel was stained with 0.5 µg/ml ethidium bromide and the figure was prepared by UV gel documentation system. Positive control *Streptococcus mutans* (ATCC 25175) (433bp) is also seen in this figure. **b:** Polymerase chain reaction (PCR) amplification of patient isolated *Streptococcus sanguinis* species in this study. Positive control (313bp) *Streptococcus sanguinis* (ATCC 10556) is also seen in this figure. **c:** Polymerase chain reaction (PCR) amplification of patient isolated *Streptococcus salivarius* species in this study. Positive control *Streptococcus salivarius* (ATCC9759) (544bp) is also observed in this figure

The primer pairs of S. mutans and S. Sanguinis, and S.salivarius were used to detect them by PCR. These primers were 5-GqaGCACCACAACATTGGGAAGCTCA-GTT and 5-GGAATGGCCGCTAAGTCAACA-GGAT for S.mutans that amplified 433bp, GGATAGTGGCTCAGGGCAGCCAGTT and GAACAGTTGCTGGACTTGCTTGTC for S.sanguinis that amplified 313bp and MKK-GTGTT-GCCACATCTTCACTCGCTTCGG and MKKCG-TTGAT-GTGCTTGAAAGGGCACCATT for S.salivarius that amplified 544bp. Blast analysis was used for determining the specificity of the sequences of candidate primers in the database (http://www.ncbi.nlm.nih.gov/Gen-Bank). The genomic isolation kit was used for isolating the genomic DNA (Thermo scientific, Lithuania), based on manufacturer instruction. DNA ladder was obtained from Cinnagen Co. (Tehran, Iran). The polymerase chain reaction (PCR) test was performed with 1µL DNA template, primer F and R (20pM) 0.7µL, DNA 0.8µL, master mix 8µL, DDW 5.8µL and 3U of LA Taq polymerase. DNA amplification was conducted in a temperature gradient thermal cycler (Biometra-T gradient, Germany) (Figure 1).

### Determination of the minimal inhibitory concentration (MIC) and minimal bactericidal concentration (MBC) Microdilution

A culture of bacteria was grown in brain heart infusion (BHI) at 37°C. 100μl of this bacterial culture was placed into the necessary number of 96-well culture plates. Using a stock solution of 640µg/ml for both plants’ extract, we prepared a series of 1:2 dilutions of them; 100µl portions were added to each well and incubated overnight at 37°C. Minimum inhibitory concentration (MIC) was determined to be the minimum concentration at which no viable cells were observed as evaluated by both microscopic examination and placing on BHI plates. In addition, minimal bactericidal concentration (MBC) has been assessed.

### Macro dilution (Tube dilution)

In this study, macrodilution method was used to determine MIC and MBC. Serial dilution (dilution by one-half) was used for preparing different concentrations of extracts in BHI broth medium. 

In order to obtain bacterial count of 10^6^ CFU/mL, the suspension was diluted. 1mL of diluted microbial suspension (1 microbial suspension: 2 culture medium) was added to the tubes containing serially diluted extract. The negative control tube contained culture medium and extract. The positive control tube contained only culture medium and microbial suspension. In addition, chlorhexidine 0.12% was added to another tube containing microbial suspension as a gold standard of antibacterial activity. After 24 hours of incubation at 37°C, growth and proliferation of microorganisms were evaluated and the MIC value of the evaluated extracts and chlorhexidine for each bacterial strain was determined and repeated in triplicate for each microorganism. MIC and MBC of different concentrations of the extracts and chlorhexidine were evaluated. MIC and MBC of each extract of P.anisum and O.vulgare were determined alone and in combination with each other against S.sanguinis and S.mutans and S.salivarius.

### Statistical analysis

The data has been assessed in SPSS version 18. The *p*< 0.05 has been considered a significant point. Repeated measurement, ANOVA and sidak post hoc test was used in this study.

## Results

Dental plaque of 60 participants (37 women and 23 men) with mean age of 4.65±1.12 years old, were collected and finally cultured and the intended bacterial species (S.sanguinis and S.mutans and S.salivarius) were determined as shown in Table 1.

**Table 1:** The patients’ isolation bacteria distribution

*S.mutans*	*S.salivarius*	*S.sanguinis*	Number of bacteria
+	-	-	15
-	+	-	3
+	+	-	4
+	-	+	3
-	+	+	2
-	-	+	5
-	-	-	10
+	+	+	18
	60		ALL

The inhibitory effect of hydroalcoholic extract of P.anisum and O.vulgare on those extracted bacteria was evaluated by macrodilution and microdilution methods and these two methods showed similar results. Based on repeated measure ANOVA and sidak post hoc test, there were significant differences between the MIC and MBC of these two extracts and their combination (*p*< 0.001).

**Figure 2:**
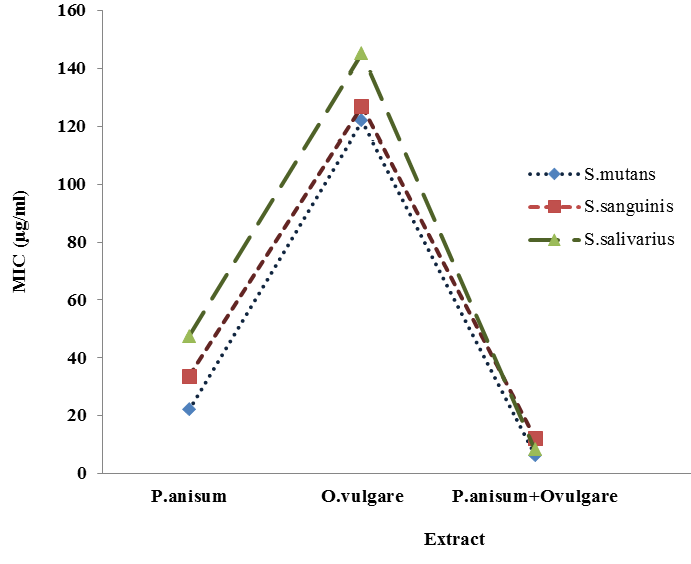
Comparison of the minimum inhibitory concentration (MIC) of the 3 extracts on 3 bacteria

**Figure 3:**
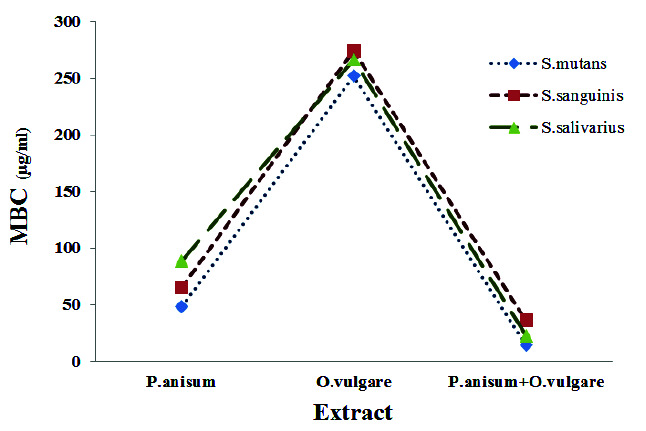
Comparison of the minimum bactericidal concentration (MBC) of 3 extracts on 3 bacteria

The mean MIC and MBC of P.anisum, O.vulgare extract, and their combination against S.mutans were statistically different (*p*<0.001). The combination of these extracts showed the most potent antibacterial properties (Table 2, Figures 2 and 3). 

**Table 2:** Mean values of minimum inhibitory concentration (MIC) and minimum bactericidal concentration (MBC) of 3 extract on 3 bacteria in 60 patients

Bacteria	*S.mutans*		*S.sanguinis*		*S.salivarius*	
Extracts	MIC (µg/ml)	MBC (µg/ml)	MIC (µg/ml)	MBC (µg/ml)	MIC (µg/ml)	MBC (µg/ml)
*P.anisum*	22.000 (SD*=13.8119)	48.750 (SD=35.0229)	33.571 (SD=16.3785)	65.714 (SD =34.365)	47.407 (SD=22.9703)	88.889 (SD=46.1880)
*O.vulgare*	122.000 (SD=98.793)	252.00 (SD=197.253)	127.14 (SD=80.040)	274.29 (SD=154.618)	145.19 (SD=93.577)	266.67 (SD=180.256)
Combination	6.4063 (SD=5.7691)	14.50 (SD=11.024)	12.32 (SD=5.69)	36.43 (SD=18.701)	8.241 (SD=4.4297)	22.96 (SD=17.827)

* SD: Standard Deviation

In addition, the mean MIC and MBC of P.anisum, O. vulgare extract and their combination against S.sanguinis were significantly different (*p*<0.001). The combination of these extracts showed the most potent antibacterial properties (Table 2, Figures 2 and 3).

In accordance with the antibacterial properties of these extracts against other incubated bacteria, the mean MIC and MBC of P.anisum extract, O.vulgare extract and their combination against S.salivarius were considerably different either (*p*< 0.001). The combination of these extracts showed the most potent antibacterial properties (Table 2, Figures 2 and 3).

**Figure 4:**
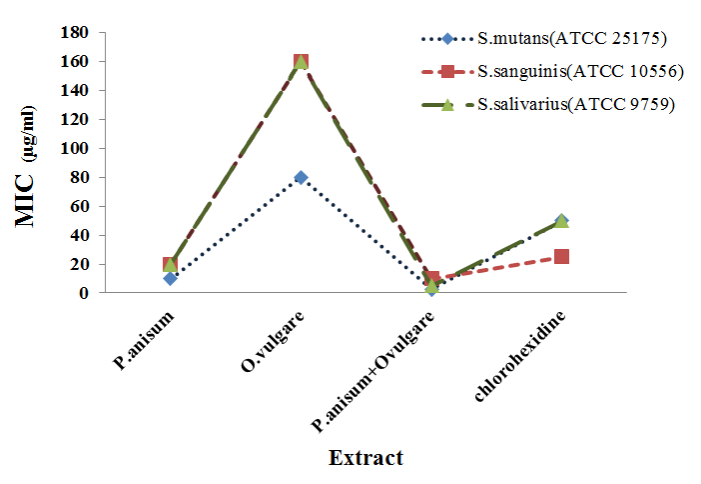
Comparison of the minimum inhibitory concentration (MIC) of 3 extracts and chlorhexidine on 3 standard bacteria

**Figure 5:**
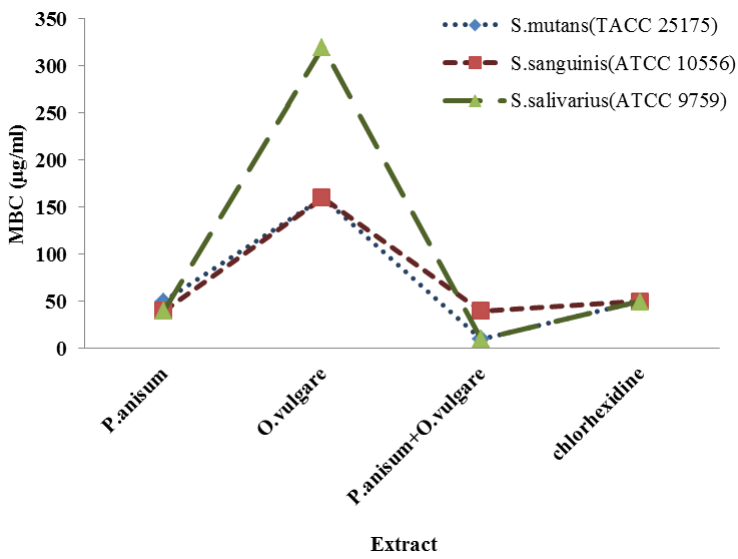
Comparison of the minimum bactericidal concentration (MBC) of 3 extracts and chlorhexidine on 3 standard bacteria

According to the findings, the most potent extract was combination of O.vulgare and P.anisum. In addition, P.anisum was more potent than O.vulgare extract. These extracts showed the best antibacterial effect on S.mutans. For better detection of antibacterial effect of the extracts, we decided to evaluate the antibacterial effect of the extracts on standard species bacteria and compare it with chlorhexidine, as a gold standard for antibacterial activity (Table 3 and Figures 4 and 5). According to these findings, a similar trend for antibacterial properties of all evaluated extracts was observed. The most potent extracts were combination of two extracts, and then P.anisum and O.vulgare extracts alone, respectively. All extracts showed the most antibacterial activity against S.mutans and exhibited lower MIC and MBC antibacterial properties than chlorhexidine except for O.vulgare. All the three species bacteria isolated from 60 patients were compared with standard bacteria. In all comparisons, the clinically isolated bacteria were more resistant to the extracts (*p*< 0.05) except for standard S.sanguinis which was more resistant than patients’ isolated species against O.vulgare extract (*p* Value for MIC<0.05). The MIC and MBC value of O.vulgare extract on S.salivarius (*p*= 0.418, *p*= 0.136) and the MBC values of the combinational extract on S.sanguinis (*p*=0.321) were not significantly different from the standard bacteria species.

**Table 3:** The minimum inhibitory concentration (MIC) and minimum bactericidal concentration (MBC) of 3 extracts and chlorhexidine on 3 standard bacteria

Bacteria	*S.mutans* (ATCC 25175)		*S.sanguinis* (ATCC10556)		*S.salivarius* (ATCC 9759)	
Extracts	MIC (µg/ml)	MBC (µg/ml)	MIC (µg/ml)	MBC (µg/ml)	MIC (µg/ml)	MBC (µg/ml)
*P.anisum*	10	20	20	40	20	40
*O.vulgare*	80	160	160	160	160	320
Combination	2.5	10	10	40	5	10
Chlorhexidine	50	50	25	50	50	50

## Discussion

Hydroalcholic extract of the P.anisum and O.vulgare was an effective antibacterial agent against S.mutans, S.salivarius and S.sanguinis so that the combination of these two extracts showed the highest antibacterial properties on all the evaluated bacteria. All extracts showed the most antibacterial activity against S.mutans and exhibited lower MIC and MBC antibacterial properties than chlorhexidine except for O.vulgare.

There are some studies on antibacterial properties of P.anisum and O.vulgare against different bacteria. Some studies have reported proper inhibitory effect of P.anisum on different bacteria with different methods [ 12 , 19 , 24 - 27 ].

Chaudhry *et al*. [ 24 ] reported antibacterial effect of aqueous extract of aniseed against S.salivarius, S.sanguinis, and S.mutans by disc diffusion method. The average diameter of the zone of inhibition of aniseed for S.salivarius was 14mm, which was the highest among these three species. In spite of more inhibitory effect of P.anisum extract on S.mutans in the present study, the report of more potent antibacterial effect of this extract against S.salivarius rather than S.sanguinis and S.mutans is considerable [ 24 ].

Kermanshah *et al*. [ 25 ] investigated the cariogenic bacterial inhibitory effect of some native Iranian plants by both broth macrodilution and agar diffusion methods. Their results about P.anisum were in the same line as those of this study. Anisum had inhibitory properties against S.mutans in all concentrations of 25, 50, 100, 200, 400µg/ml. Their results for MIC and MBC for anisum against S.mutans were 12.5µg/ml and 200µg/ml. These values are 22µg/ml (MIC) and 48.75µg/ml (MBC) for S.mutans in our study. Different values can be related to diversities of plant extraction concentration, various methodologies, and differences in cultivated geographic area of plants.

Using different solvents exhibit distinct antibacterial potency, while in a study the alcoholic extract exhibited significant inhibitory properties [ 27 ], while another evaluation reported the reverse [ 26 ]. Similar to the results of our study and the same as P.anisum, there are some reports on antibacterial properties of O.vulgare [ 12 , 17 - 19 , 28 - 33 ].

Sarac *et al*. [ 17 ] extracted the essential oils of O.vulgare by hydrodistillation, which showed effective antibacterial properties against gram positive and negative bacteria. Inhibition zone of O.vulgare against S.mutans was 19mm, which was more than some antibiotics such as penicillin (15mm), Ampicillin (12mm) and Cefoperazone (14mm). Some studies listed the biochemical composition of O.vulgare and determined carvacrol and thymol as the most effective ingredients of this plant against bacteria [ 12 , 18 , 28 - 31 , 33 ]. In addition, Khan *et al*. [ 28 ] regarded O.vulgare as a green alternative to control dental caries. They showed a significant decrease in viability, metabolic activity, and biofilm formation of S.mutans by thymol and carvacrol. Even a study introduced these two extracts as a more potent antimicrobial agent than beneficial probiotic bacteria (Lactobacillus) [ 12 ].

A research exhibited this potency in comparison to traditionally used essential oil in the dentistry clove oil [ 31 ]. The present study revealed proper antibacterial effects (bacteriostatic/bactericidal) for O.vulgare, though its MIC and MBC like other studies were relatively high and more than P.anisum values.

Magi *et al*. [ 25 ] reported a range of MIC between 256-512µg/ml for essential oil thyme of Oregano and 64-250µg/ml against Group A Streptococci for carvacrol. Although these values were high for MIC, they can be indicative of antibacterial effects in accordance with the finding of this study; MIC for S.mutans, S.sanguinis and S.salivarius was 122, 127, 145µg/ml. In this regard, Ozkalp *et al*. [ 32 ] discussed the strong antibacterial activity of O.vulgare oil and its higher activity against Gram-positive bacteria. The findings of our study have confirmed the general results of aforementioned studies. 

In addition to antibacterial activity of P.anisum and O.vulgare, the noticeable synergistic activity of these two extracts in combination with each other is very practical and usable.

Different plants with distinct compositions show various properties with diverse degrees of antibacterial activities. Diversities of geographical areas of plants and their different climates play a very important role in determining the dominant composition of plants. For example, Saudi Origanum oil is carvacrol dominant, but Jordanian Oraganum is thymol dominant [ 25 ].

In the literature, antibacterial activity of different O.vulgare extracts has been assumed to be related to essential oils, flavonoids and triterpenoids. Essential oils contain a high percentage of phenolic ingredients including carvacrol, eugenol, E2-methoxy-4- (2-propenyl) phenol and thymol which are the most potent antibacterial agents [ 34 - 35 ].

In addition to some other properties of thymol and carvacrol, such as immunity enhancement against virus and tumor and anticancer activities, some mechanisms for their antibacterial activity have been proposed. These essential oils permeabilize and depolarize the cytoplasmic membrane, which causes a decrease in pH and consequently disturbs the proton motive force, reduces the intracellular ATP level, and finally causes cell death [ 28 , 30 ].

Carvacrol and thymol mediated cell lysis can also be related to over-expression of autolysin gene, which can affect the cell wall [ 31 ]. In addition, down-regulation of glycosyl transformase B gene, mediated by thymol and carvacrol, justified their antibiofilm properties [ 36 ].

The selection of a relevant method for evaluating the antibacterial properties is important. Confounding factors, which can affect disk or agar diffusion method, are more prominent in broth dilution method. Chemical composition of essential oils, their agar diffusion rate, and chemical volatility can affect the size of inhibition zone. Different solvents with various polarities can extract diverse compositions. Compatibility of the solvent’s polarity and plant composition to extract all ingredients as much as possible should be considered. Since thymol and carvacrol are not water soluble [ 37 ], a hydroalcoholic solvent was selected for the present study.

Except for thymol and carvacrol, other ingredients of O.vulgare, such as α-pinen, terpinene, β-pinen, can destroy the cytoplasmic membrane structure and blocking electron transport. Other compositions like linlool, terpinen-4-ol and terpineol denature proteins and solve or dehydrate the cells [ 18 ].

Some important compositions of aniseeds essential oil are transanethole (84-94%), estragol, γ-hymachalen, panisaldehyde and methyl chavicol [ 34 ], Transanthole, methyl chavicol, linalool, anisaldehyde, limomene, α-pinene, methyl eugenol, and bomeol [ 19 ].

According to what European Scientific Cooperative on Phytotherapy (ESCOP) stated, anethol, β-caryophyllene and flavonoids are effective compositions against dental caries responsible bacteria, especially *S.mutans* [ 25 ]. Kermanshah *et al*. [ 25 ] indicated that the hydrophilic agents of P.anisum are responsible for antibacterial effect. 

Selecting proper solvent, evaluating the synergistic activity of using two effective extracts and using macrodilution and microdilution methods for evaluation to use patient isolated bacteria instead of standard species are the strong points of our research. Studying the patient-isolated bacteria can emerge more practical horizons for introducing antimicrobial agents for resistant bacteria.

On the other hand, introducing a new combinational powerful green and natural antibacterial agent for decreasing dental caries bacteria can be considered for commercial usage, concerning antibiotic resistance and some side effects of public mouthwashes. These extracts or their effective essential oils can be used in mouthwashes and toothpastes to control bacterial growth and biofilm formation.

Selecting accurate methods for evaluating antibacterial properties, studying other solvents, performing GC-MS analysis on Iranian P.anisum and O.vulgare, and determining the most effective ingredients of these plants can be considered for future studies.

## Conclusion

The hydroalcholic extract of P.anisum and O.vulgare on patient isolated dental caries bacteria (S.mutans, S.sanguinis, and S.salivarius) showed proper antimicrobial properties. The combination of these extracts exhibited very significant potency in antibacterial activity.

## Conflict of Interest

The authors declare that they have no conflict of interest.
